# Respiratory response to temperature of three populations of *Aurelia aurita* polyps in northern Europe

**DOI:** 10.1371/journal.pone.0177913

**Published:** 2017-05-17

**Authors:** Danja P. Höhn, Cathy H. Lucas, Sven Thatje

**Affiliations:** National Oceanography Centre Southampton, University of Southampton, Southampton, United Kingdom; University of Connecticut, UNITED STATES

## Abstract

The benthic life stage (polyp or scyphistoma) of the bloom-forming jellyfish, *Aurelia aurita* (Linnaeus, 1759), also known as the moon jellyfish, contributes to the seasonal occurrence and abundance of medusa blooms via asexual reproduction. *A*. *aurita* is widely distributed in coastal areas in northern Europe, and one of the most studied jellyfish species. While the physiology of the visible medusa is largely understood, understanding of the physiology of the perennial benthic life-stage is scarce. To measure the physiological tolerance of *A*. *aurita*, the scyphistoma’s temperature sensitivity across its distributional range was investigated. Respiration rates of polyps from three northern European locations exposed to 11 temperatures between 2 and 22°C were measured. There was a significant difference in respiration rate among the three polyp populations, which may reflect on differences in their thermal tolerance window. A critical temperature was reached at 14°C with the metabolic rate decreasing below and above that temperature. This pattern was less pronounced in the Norwegian population but polyps were able to survive, at least temporarily, those temperatures exceeding their natural range. While polyps collected from northern Norway, with a narrow environmental thermal window, displayed a low baseline metabolism with a Q_10_ value of 1.2, polyps from southern England and Scotland had Q_10_ values of 1.6 and 2.5, respectively. Differences in polyps’ respiration rates across their distributional range suggest that populations have evolved adaptations to local environmental thermal conditions.

## Introduction

Jellyfish blooms have become a concern in many regions of the world. Blooms have socio-economic impacts on fisheries and aquaculture, coastal tourism and power stations [1]. Furthermore, bloom events have the potential to affect ecosystem structure and function through changes in top-down control [2,3], trophic pathways [4], and biogeochemical cycling including organic flux to the sea floor [5,6]. Thus, they are the focus of concerted research efforts into understanding the causes of bloom events. The study species, *Aurelia aurita* is found in northern Europe as shown by Dawson et al. [7,8]. *A*. *aurita* populations display differences in abundance and life history dynamics over large spatial and temporal scales, which makes predictions of future bloom dynamics difficult [9].

The polyp life stage is critical in ensuring the successful recruitment and maintenance of jellyfish populations. It is thought to be a key factor in the formation of jellyfish blooms because of its ability to reproduce asexually in various ways [10]. Mechanisms and rates of asexual reproduction have been increasingly studied over the last ~10 years. Temperature is a principal environmental controller of the life-cycle of scyphozoans, including survivorship, growth and reproduction in polyps [11– 14]. While it is known that temperature induces various forms of asexual reproduction such as budding, strobilation, and the formation of podocysts [14–16], information about the functional biology of the polyp is largely unavailable. To our knowledge, there is one experimental study that has investigated the effect of temperature on the oxygen consumption of *Aurelia aurita* polyps in northwestern Europe [17]. Examining the limits of temperature tolerance in polyps of bloom-forming jellyfish species will help us to predict the scale of recruitment of new medusae and thus the potential size of jellyfish populations.

While reproduction represents one way of measuring fitness, respiration provides an indirect way of measuring acute changes in metabolic rates of polyps in response to temperature variation. In ectotherms such as scyphozoan polyps, a temperature increase accelerates most physiological processes, including the rate of oxygen consumption within the temperature range an animal can tolerate. As a general rule, a rise of 10°C increases the rate of oxygen consumption by twofold to threefold—called the Q_10_. If temperatures are too high, Q_10_ values may be less than one, indicating a loss of function. At low temperatures, Q_10_ values may be much greater than one possibly indicating that energy barriers and activation energy are increased.

Living within estuarine and coastal environments, many scyphozoan medusae display high tolerance to environmental change including changes in salinity and temperature [9]. Thermal tolerance limits within a species are the result of the environmental temperature range at a given geographic location that can change over time, so that adaptation (selection for a distinct genotype) to a specific acclimation temperature becomes possible. However, exceptions exist and a different degree of tolerance to extreme temperatures may occur during ontogeny or certain periods of their life-cycle. The scyphozoan *A*. *aurita* is widely distributed in coastal, estuarine, and brackish-water environments of northern Europe [7,8] and is able to tolerate large and frequent temperature fluctuations typical of shallow coastal environments.

The 2°C temperature increase in sea-surface temperature associated with global warming that is predicted for the end of the 21^st^ century (IPCC report 2013, RCP8.5) will expose populations to increased thermal stress. Exploring the thermal tolerance range of aquatic metazoans, including jellyfish, will increase the chance of realistic predictions of population size. Differences in respiration rates between geographic habitats can be used to indicate physiological tolerance in populations of *A*. *aurita* within northern Europe.

Several studies have examined oxygen consumption in medusae, but only few have measured respiration rate in polyps [17–19]. The aim of this study is to examine the respiratory response to temperature of *A*. *aurita* polyps taken from three different populations within northern Europe. It is hypothesised that *A*. *aurita* inhabiting different geographic locations respond differently to temperatures, depending on the seasonal temperature range they experience. This will provide important insight into the metabolic rate of *A*. *aurita* polyps and examine the effects of latitudinal adaptation in a widely distributed coastal species.

## Materials and methods

### Establishing polyp cultures

**Fig 1:**
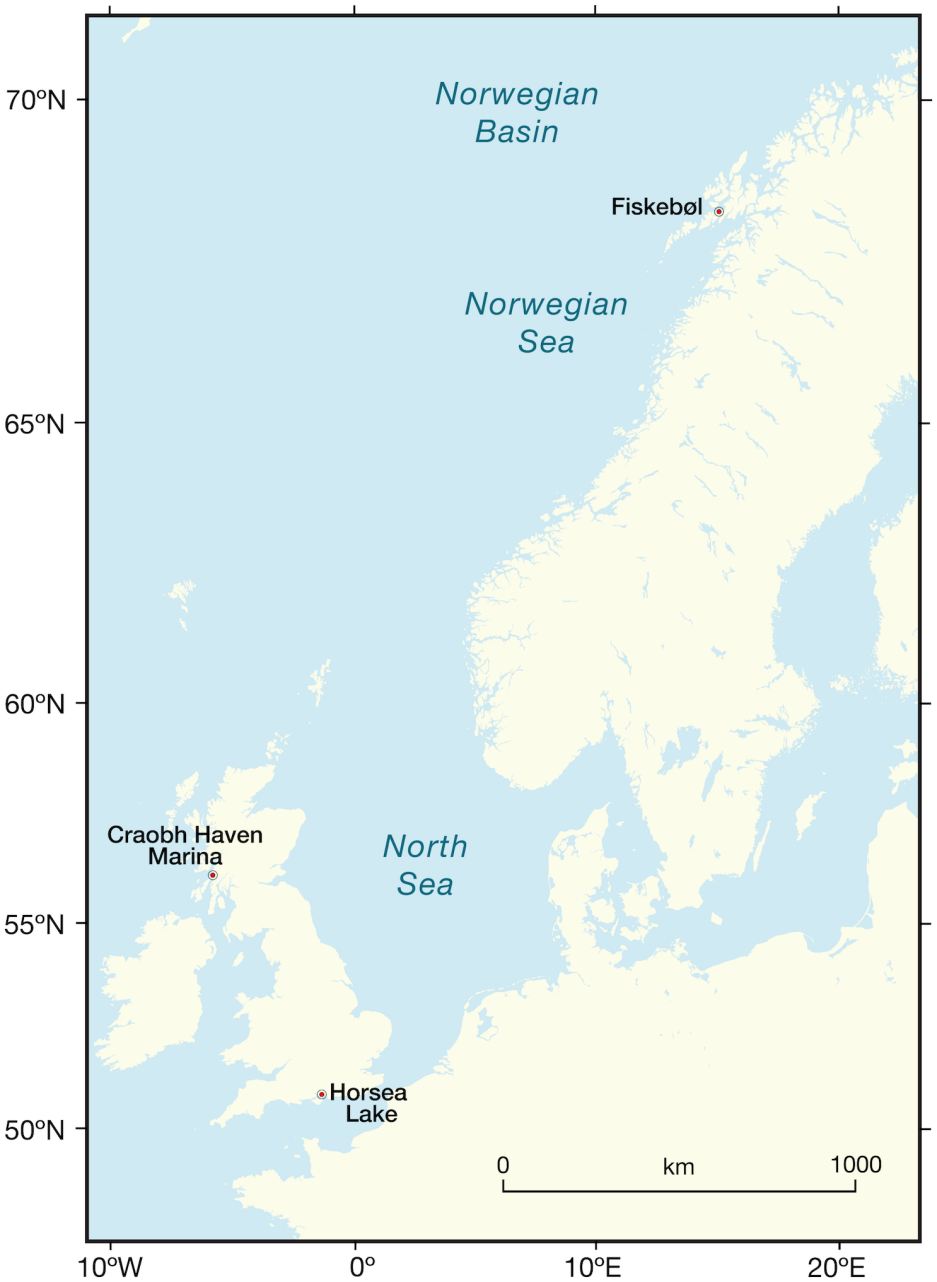
Map of northern Europe showing the locations of the three populations of planula-larvae bearing *Aurelia aurita* medusae that were used in this study: Southern England (Horsea Lake), Scotland (Craobh Haven) and Norway (Fiskebøl). Reproduced under a CC BY license by permission of Kate Davis, School of Ocean and Earth Science, University of Southampton.

**Fig 2:**
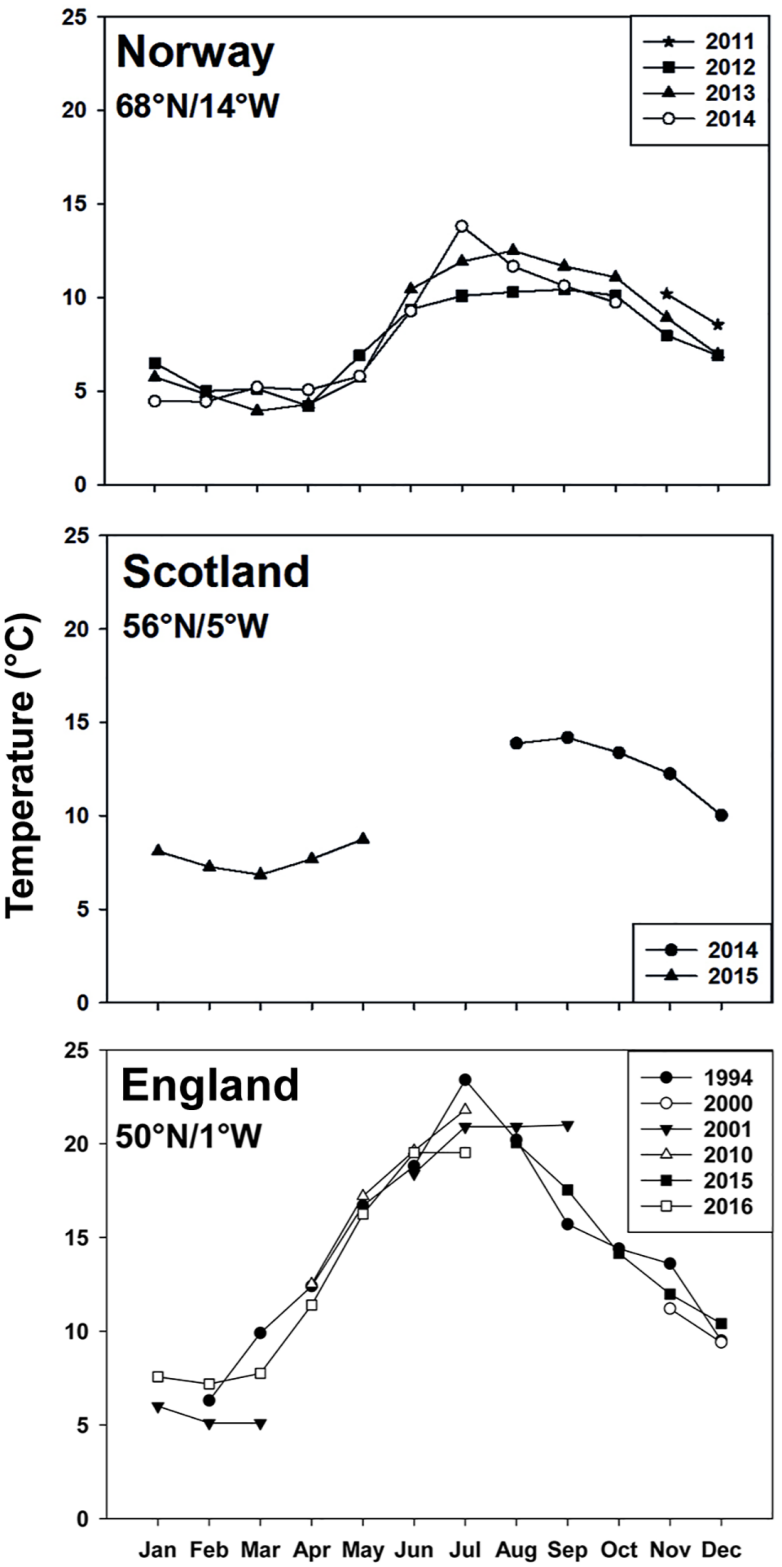
Mean monthly water temperatures at Eggum (Norway), Dunstaffnage (Scotland) and Horsea Lake (southern England) where *Aurelia aurita* were collected. Horsea Lake temperature data were taken from [20]. Norwegian temperature data were received from the Institute of Marine Research—Norwegian Marine Data Centre (NMD) and temperature data from Scotland were received from the Scottish Association for Marine Sciences (SAMS).

The following three populations of *Aurelia aurita* (Linnaeus, 1759) were studied (Fig 1): Horsea Lake, an enclosed brackish water lake on the south coast of England (50° 83’ 68.26” N / 1° 10’ 19.11” W) HMS Excellent Royal Navy provided permission; Craobh Haven marina, Argyll, in Scotland (56° 21’ 12.79” N / 5° 55’ 67.63” W) no permission was required; Fiskebøl, in northern Norway (68° 43’ 12.92” N / 14° 82’ 53.31” W) no permission was required and the field studies did not involve endangered or protected species. (To the best of our knowledge, all populations studied here are within species’ native range [8]). Reproductively ripe female medusae containing planula larvae were randomly collected with a scoop net from Horsea Lake in June (Fig 2). Medusae were transported back to the research aquarium at the National Oceanography Centre, Southampton (NOCS) in temperature-controlled boxes filled with ambient seawater (14°C). Oral arms of medusae were gently rubbed to release brooded planula larvae. Petri dishes were placed on the surface of each container, for the planula larvae to settle on and metamorphose into polyps. In northern Norway ripe female *A*. *aurita* medusae were collected at Fiskebøl ferry terminal in June. Oral arms of medusae were gently rubbed to release planula larvae into a small container filled with ambient seawater (14°C), which was transported back in an insulated container to NOCS within two days. In Scotland, ripe medusae were collected at Craobh Haven Marina, Argyll in June (14°C) and transported back to the Alan Ansell Research Aquarium at the Scottish Association for Marine Science (SAMS); larvae were settled out as described above before being transported in temperature controlled containers to the NOCS.

When planula larvae had metamorphosed into polyps, the containers were moved into temperature controlled 12°C ± 0.5°C (salinity 32) incubators and maintained, well aerated, in darkness for four months. *A*. *aurita* polyps were fed two-day old *Artemia* nauplii in excess five times a week and water was changed before food was added. Polyps were exposed to eleven different temperatures, 2°C, 4°C, 6°C, 8°C, 10°C, 12°C, 14°C, 16°C, 18°C, 20°C and 22°C, capturing the full range experienced by polyp populations from southern England and exceeding the range of polyps from Scotland and Norway in their natural habitat. Polyps experienced a temperature change of 2°C per week, starting at their acclimation temperature of 12°C. Each population was divided into two groups, with one group acclimating upwards and the second group acclimating downwards. During temperature acclimation, polyps were kept in full darkness, except for feeding and water changes (30 min d^-1^). Dark conditions were chosen to minimise algae growth and mimic their natural habitat. For each treatment, handling time was kept to an absolute minimum and the conditions of polyps were monitored throughout.

### Respiration versus temperature experiments

Experimental polyps were last fed 24 h prior to respiration measurements, and only healthy polyps with fully developed and extended tentacles were chosen from the experimental culture. Individual polyps, of similar size, were monitored and carefully detached from each experimental container using a dissection knife before being placed into the respiration chamber (1 polyp per chamber). Healthy polyps were released into an aquarium with 32 salinity, 1μm-filtered and UV treated seawater acclimated to the experimental temperature, and were then recaptured and placed in 2.8 ml plastic vials. To prevent the formation of an oxygen saturation gradient within the vial a small magnetic stirrer was placed at the bottom of each vial. A fine mesh was placed within the bottom half of the vial, for the polyp to rest on and to ensure separation of the polyp from the magnetic stirrer. The vials were sealed underwater to prevent air bubbles being trapped on the inside. The sealed vials were kept upright in a water bath for the incubation period. The water bath temperature was controlled by a chiller and heater system (model: HAAKE DC1 K15) (±0.5°C) throughout the experiment. The water bath temperature was also monitored using a thermometer placed into the water bath. The vial was monitored during the experiment to ensure that the seawater oxygen concentration within the vial did not drop below 60% saturation, to eliminate potential effects of hypoxia [21]. The incubation time was 4 h for each temperature treatment and experiments started at 09:00 h. Following the incubation period, the oxygen saturation of seawater inside the vial was measured using a Presens Microx TX 3 temperature-adjusted oxygen meter and microoptode [22]; the microoptode was calibrated daily. The microoptode within a hypodermic needle was held in place using a clamp and stand and the optode was immediately inserted into the experimental vial once opened. Oxygen concentration (μmol l^-1^) was calculated for 100% oxygen-saturated seawater under the conditions (e.g. 32 salinity, 2.8 ml volume vial, 4 h incubation and temperature) used in the experimental treatments [23]; three controls were provided. Following the respiration experiments polyps were washed in distilled water; blotted dry on paper and then transferred into pre-weighed 6x4 mm tin-capsules. Samples were freeze-dried for 16 h, using a freeze drier (Thermo Scientific Heto PowerDry LL33000), and dry weights (DW) were measured using a microbalance (Sartorius ME5). To calculate the oxygen consumption of each polyp, the difference in final oxygen concentrations between the controls and experimental vials were calculated to correct for microbial respiration. Respiration rates (nmol O_2_ h^-1^) were calculated by including salinity, temperature, the incubation period (time in min) and the volume of the vial [22]. For the weight specific respiration rate (nmol O_2_ mg DW^-1^ h^-1^) the DW (mg) was included. The sample size of each population was: England n = 54, Scotland n = 79 and Norway n = 81. In the Scottish population one temperature is missing due to probe failure.

### Statistical analyses

Statistical analysis was carried out using R. An ANCOVA was performed on the respiration rates (as nmol O_2_ h^-1^) of the three *Aurelia aurita* polyp populations (Table 1). Location and water temperatures were used as factors and dry weight was used as a covariate in the model. Both factors (temperature and location) and weight showed a highly significant effect on the respiration rates (nmol O_2_ h^-1^) of polyps.

**Table 1:** Results of the ANCOVA. Temperature and location were used as factors and dry weight as a covariate in the model.

	Df	Sum Sq	Mean Sq	F value	Pr(>F)
**RR (nmol O_2_ h^-1^)**					
**Weight**	1	1316.75	1316.75	162.30	<0.0001 ***
**Factor (Temperature+Location)**	18	2008.00	111.56	13.75	<0.0001***
**Residuals**	112	908.64	8.11		

Signif. codes: 0 ‘***’

Mean respiration rates of the three populations were compared with a One-Way ANOVA and Holm-Sidak post hoc test.

## Results

### Respiration rates versus temperature

**Fig 3:**
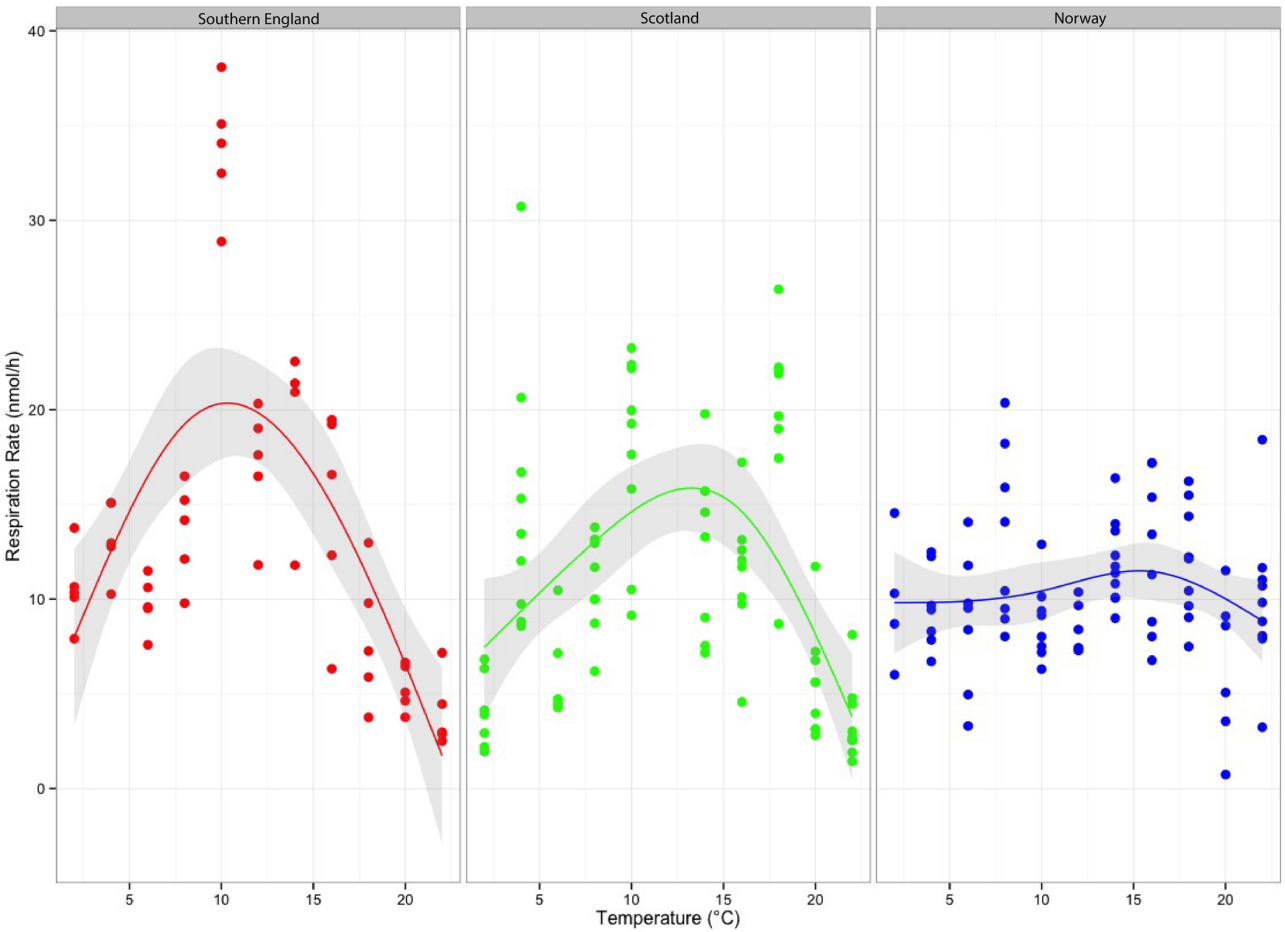
Respiration rates (nmol O_2_ h^-1^) of *Aurelia aurita* polyps versus temperature (2–22°C). Three populations are compared: southern England (n = 54), Scotland (n = 79) and Norway (n = 81). Scatter plot plus smoothing curve (shaded area = residuals)—a line that represents the data but does not go through each data point—is displayed. Data were analysed in R. Data are available in S1 Dataset in supplementary materials. A plot of weight-specific RR vs temperature is also available in electronic supplementary materials (S3 Fig).

Overall, *Aurelia aurita* polyps’ respiration rates ranged from a minimum of 1 nmol O_2_ h^-1^ to a maximum of 38 nmol O_2_ h^-1^ across the different temperatures (Fig 3). Respiration rates (nmol O_2_ h^-1^) increased linearly with polyp weight with a linear regression of R^2^ = 0.70 (P<0.05, see S2 Fig and statistics in supplementary materials). Furthermore respiration rates were significantly different between locations and temperatures (ANCOVA: F_19,112_ = 21.57, P<0.0001) (Table 1). The three *A*. *aurita* populations showed a different response in respiration rates over the temperature range from 2 to 22°C (One-Way ANOVA: F_2,211_ = 4.17, P<0.01). The mean respiration rates of southern England polyps were significantly higher compared to polyps from Scotland (Holm-Sidak: P = 0.02) and Norway (Holm-Sidak: P = 0.01). The results of a within-location multiple comparison can be found in the supplementary materials (S2 Dataset).

Respiration rate patterns differed among all three populations, with the greatest changes in respiration rates over temperature observed in the Scottish polyps (< 1 to 36 nmol O_2_ h^-1^) and in the southern England polyps (3 to 38 nmol O_2_ h^-1^). The smallest changes in respiration rates were observed in the Norwegian polyps (< 1 to 20 nmol O_2_ h^-1^) as well as the lowest mean respiration rate (Fig 3).

The respiration rates of *A*. *aurita* polyps from southern England and Scotland increased between 2 and 14°C by Q_10_ value of 1.6 and 2.5, respectively. The respiration rates of the Norwegian polyps increased by a Q_10_ value of 1.2 between 2 and 14°C. After 14°C the respiration curves decreased to 22°C with a Q_10_ value of 0.14 in the southern England and with a Q_10_ value of 0.21 in the Scottish population. The Q_10_ value (0.8) in the Norwegian population was similar between 14 and 22°C (Fig 3).

## Discussion and conclusions

All organisms live within a limited range of temperatures, adjusted to all eco-physiological processes including coordination and function of molecular, cellular, and systematic cycles [24,25]. Thermal tolerance windows are as narrow as possible within their associated environment to minimise maintenance costs, resulting in functional differences in populations from different latitudes [26]. The current experiment was carried out in order to examine physiological bottlenecks and differences in *Aurelia aurita* polyp populations, in terms of its respiratory response to a series of temperatures. Differences in respiratory response were observed between *A*. *aurita* polyps from three different geographic locations in northern Europe, indicating acclimation to local sea temperatures.

An increase in respiration rates between 2 and 14°C with Q_10_ values of 1.6 and 2.5 were observed in southern England and Scottish polyps. Similarly, Q_10_ values of 2.5 and 1.8 have been reported for Antarctic marine ectotherms and epipelagic marine zooplankton [27,28]. This indicates that the thermal effects measured here were within the species’ normal range, as for many biological processes including respiration rates and enzymatic activity, Q_10_ values near two are observed [29]. At temperatures greater than 14°C respiration rates decreased, with Q_10_ values of 0.14 in the southern England population and 0.21 in the Scottish population, indicating that their performance had decreased rapidly after 14°C and up to a maximum temperature of 22°C, at which all biological functions ceased. Similarly, Mangum et al. [18] observed Q_10_ values <1.00 in *A*. *aurita* polyps at warmer temperatures. At high temperatures lethal effects may occur with Q_10_ values less than one, indicating that increases in temperature are damaging the system in question—even leading to irreversible loss of function [29].

Furthermore, the bell-shaped metabolic response to temperature could have been influenced by acclimation time (weekly temperature changes of 2°C) and duration of captivity with each 2°C change. Thermal sensitivity—the ability of an organism to withstand a range of temperatures—can be shifted by acclimation time, as well as the actual temperature. While higher reproductive rates have been observed at warmer temperatures in polyps from the Mediterranean and Baltic Sea [13,14], other studies have found lower rates in polyps from Taiwan and the Mediterranean Sea [30,31]. These findings suggest that thermal optima can differ, suggesting site- and population-specific adaptation [32].

*A*. *aurita* polyps collected from southern England experience a large annual temperature range from a minimum of 6°C in winter to a maximum of 23°C in summer (Fig 2) which might explain the large differences in respiration rates with temperature. The Scottish population experiences temperatures between 4°C and 14°C (Dunstaffnage, Fig 2), and this might explain the steep decline in respiration rates at temperatures above 14°C.

Gambill and Peck measured respiration rates in scyphozoan polyps from the Baltic Sea and NE Atlantic and found similar rates to our study (following conversion for comparative purposes) [17] (Table 2). For this conversion, the dry weight of the Baltic Sea polyps was standardised to a dry weight at salinity 32, on the assumption that the salt content of the polyp is the same as ambient salinity (Baltic Sea, 20 salinity) [33].

**Table 2:** *Aurelia aurita* and *Cyanea capillata* polyp oxygen consumption at different temperatures. The respiration rates of *A*. *aurita* and *C*. *capillata* polyps from the Baltic Sea have been standardised to a DW at salinity 32, to remove the effect of salinity on weight [3,3,3,4]. Data were adapted from [1,7].

Species (μg)	Temperature (°C)	Oxygen consumption (nmol O_2_ mg^-1^ h^-1^)	Location
***A*. *aurita*** (500)	7	5.41	Baltic Sea
	10	4.69	
	12	4.69	
	15	10.10	
	18	11.95	
	20	9.19	
***A*. *aurita*** (525)	7	5.56	North East Atlantic
	10	5.46	
	12	13.32	
	15	11.69	
	18	12.25	
	20	6.85	
***C*. *capillata*** (500)	7	5.95	Baltic Sea
	10	5.65	
	12	6.49	
	15	11.77	
	18	11.59	
	20	10.86	

Oxygen consumption data of polyps were derived from Fig 2 in [17]. Data points were measured by the use of Photoshop, then converted into common metrics of nmol O_2_ mg^-1^ h^-1^ to be comparable with the current study. DWs of polyps from the Baltic Sea have been converted to DWs at salinity 32. All dry weights (μg) are given below each species name.

While respiration rates of southern England and Scottish polyps peaked at 14°C, NE Atlantic polyps peaked at 12°C, and Baltic Sea polyps peaked at 15 and 18°C. All rates were lower at temperatures above and below these peaks. Temperatures >15°C are most likely out of the normal range of Baltic Sea polyps as *in situ* temperatures from the region range between 3 and 15°C, while NE Atlantic temperatures range between 8 and 14°C [17]. Although dry weights have been standardised, we acknowledge it might not be the best metric for characterising mass in polyps as it may change with salinity [33,34]. Nevertheless, respiration rates in both studies scaled allometrically with weight (value of 0.7 for interspecific regression) and did not increase exponentially with temperature as observed in medusae [35].

In contrast to the u-shaped response curve observed in our work and in previous studies of polyps, respiration rates in *A*. *aurita* medusae and ephyrae have been observed to increase exponentially with temperature. The respiration rates in *A*. *aurita* medusae from Skive Fjord, Denmark increased between 7 and 22°C with a higher Q_10_ value of 3.1, while the respiration rates of ephyrae increased between 11.5 and 22°C [35]. It seems that polyps of *A*. *aurita* might be less tolerant of warmer temperatures, because their respiration rates ceased at 14°C (with a lower Q_10_ value than medusae) in the present study and at 12°C in the NE Atlantic polyps [17]. Metabolic rates among jellyfish life-stages have been suggested to scale isometrically with the lowest respiration rates in polyps compared to those of ephyrae and medusa [17,35,36]. Differences are most likely caused by a different morphology, diet and activity level as polyps hardly move [37].

Norwegian polyps experience a much cooler temperature range, from 3 to 14°C, compared to those from southern England (4–23°C) (Fig 2). The respiration rates of Norwegian polyps showed a small increase but there was little change across all measured temperatures, suggesting a lower capacity to respond physiologically to temperature variability [38]. A low Q_10_ value in the Norwegian population may indicate that temperatures were too high, especially after 14°C, causing damage to the biochemical respiration system and leading to a loss of function [29].

Overall, the three populations that were tested responded differently across the same temperature range, although all were maintained at the same conditions (12°C, 32 salinity) for about 4 months prior to the experiment. For future experiments it may be useful to measure anaerobic metabolism, as critical thermal limits are usually associated with a decrease in aerobic metabolic processes and an increase in anaerobic processes [24,38]. Some invertebrates use metabolic depression, anaerobic energy production, and stress protection mechanism to provide short- to medium- term tolerance to extreme temperatures [24,39]. Respiration rates differed significantly between polyps originating from higher and lower latitudes, which may be related to a variation in their thermal tolerance window, since latitudinal changes are most likely driven by environmental temperature [24]. Temperatures in southern England range from 4 to 23°C, in Scotland from 4 to 14°C and in Norway from 3 to 14°C. Similarly, geographic differences in the effect of temperature on survival and asexual reproduction of *A*. *aurita* s.l. polyps has been observed among populations from the Mediterranean Sea, Baltic Sea and the Red Sea [12]. The authors found that *A*. *aurita* polyps could maintain their reproductive features even under different environmental conditions, suggesting acclimation to their natural environment. The latitudinal differences between the three polyp populations studied here, suggest thermal adaptation to polyps’ natural environment [32]. Polyps originating from higher latitudes (Scotland and Norway) experience a much narrower temperature range compared to those from southern England, but—and to our surprise—were able to maintain respiration rates at temperatures exceeding their current environmental thermal range, at least in the short term of the experiment. Nevertheless, polyps from southern England and Scotland had decreased respiration rates above 14°C indicating a sensitivity to higher temperatures. Polyps might be able to compensate in the short term for temperature changes, but differences in polyps’ respiration rates across their distributional rage suggests adaptation to local thermal conditions. Thus, a 2°C temperature increase in response to global greenhouse gas emission may be challenging for polyps.

## Supporting information

S1 Dataset Complete set of data used in analysis. (XLSX)

S2 Dataset Post-hoc results of the within location comparison. (XLSX)

S1 Fig Photograph of *Aurelia aurita* polyps. With permission from Matt Doggett to publish under a CC BY license.
(TIF)

S2 Fig Respiration rate (nmol O_2_ h^-1^) versus weight (mg) of *Aurelia aurita* polyps expressed as a linear regression line. There was a significant linear relationship between respiration rate and dry weight at nine temperatures (linear model: P < 0.05) with the following R^2^ values: at 2°C, R^2^ = 0.56; at 4°C, R^2^ = 0.45; at 8°C, R^2^ = 0.24; at 10°C, R^2^ = 0.44; at 14°C, R^2^ = 0.89; at 16°C, R^2^ = 0.88; at 18°C, R^2^ = 0.73; at 20°C, R^2^ = 0.70 and at 22°C, R^2^ = 0.67.
(TIF)

S3 Fig Respiration rates (nmol O_2_ mg DW^-1^ h^-1^) versus temperatures. Three populations are compared: southern England (n = 54), Scotland (n = 79) and Norway (n = 81). Scatter plot plus smoothing curve (shaded area = residuals)–a line that represents the data but does not go through each data point—is displayed. Data were analysed in R. Data are available in electronic supplementary material (S1 Dataset).
(TIFF)
