# Supplementary figures and images for: Respiratory response to temperature of three populations of Aurelia aurita polyps in northern Europe
Source: PLoS One. 2017 May 17;12(5):e0177913. doi: 10.1371/journal.pone.0177913 (PMC5435318; doi:10.1371/journal.pone.0177913)

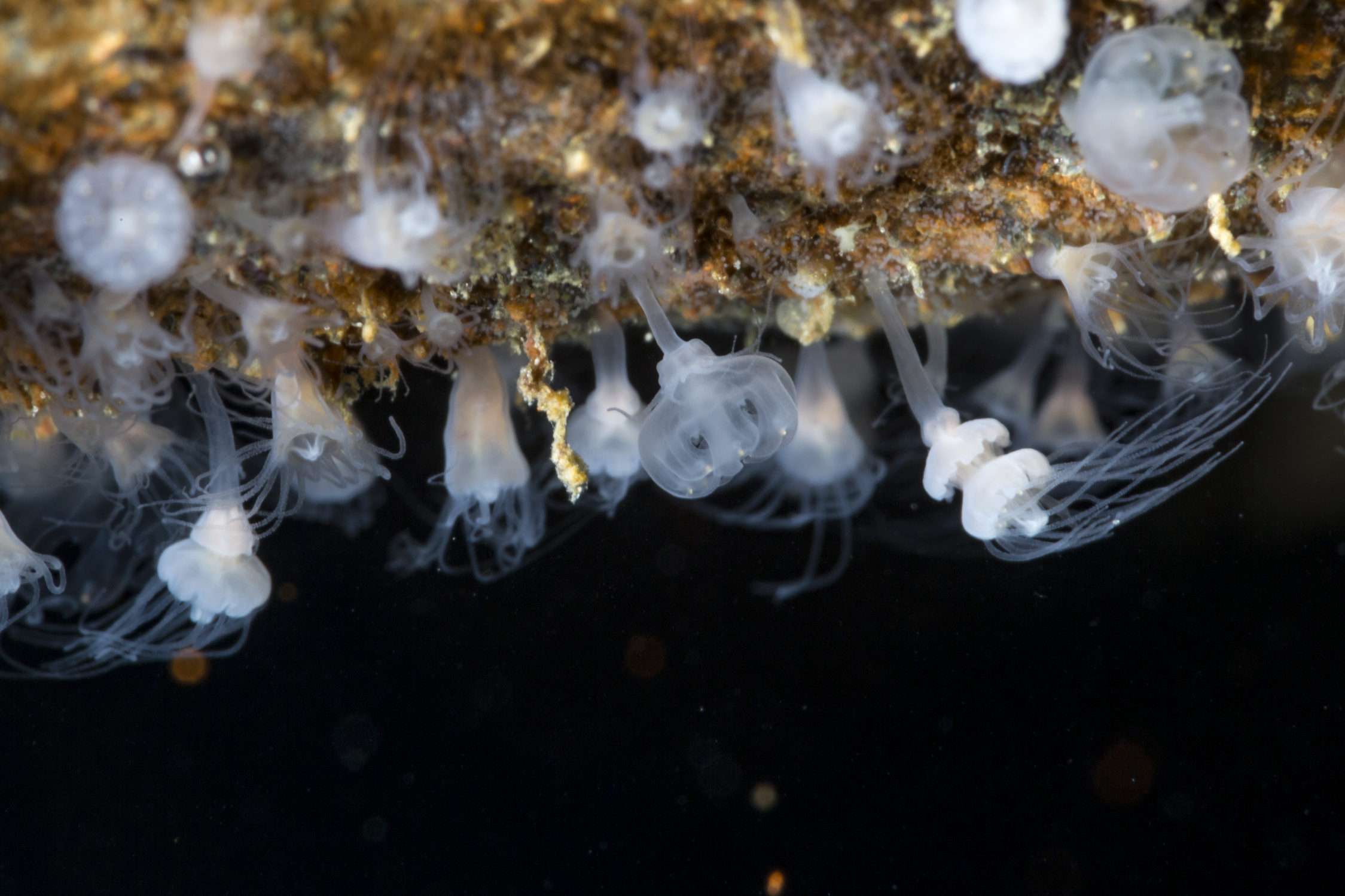

Supplement: S1 Fig — With permission from Matt Doggett to publish under a CC BY license. (TIF) [file pone.0177913.s003.tif]

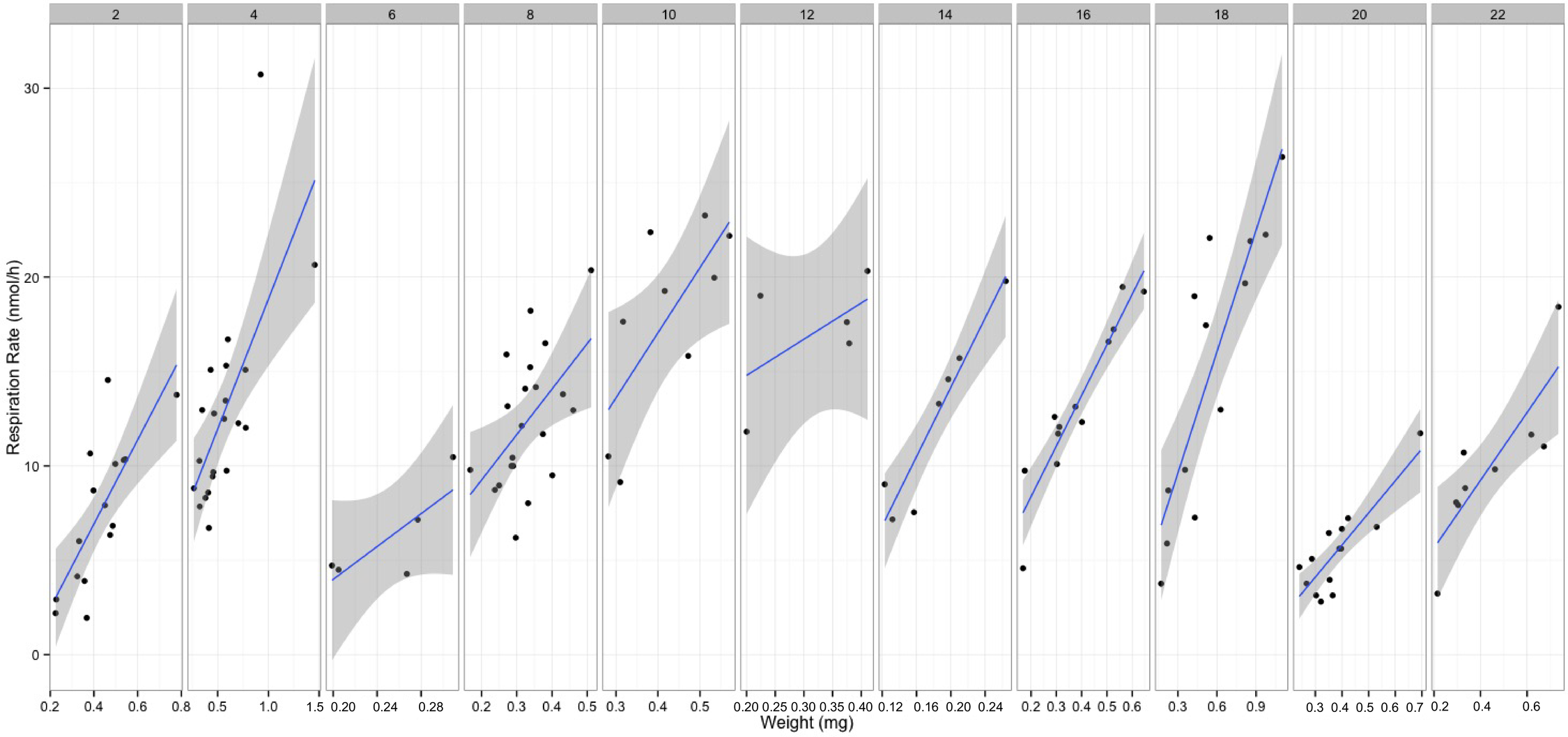

Supplement: S2 Fig — There was a significant linear relationship between respiration rate and dry weight at nine temperatures (linear model: P < 0.05) with the following R2 values: at 2°C, R2 = 0.56; at 4°C, R2 = 0.45; at 8°C, R2 = 0.24; at 10°C, R2 = 0.44; at 14°C, R2 = 0.89; at 16°C, R2 = 0.88; at 18°C, R2 = 0.73; at 20°C, R2 = 0.70 and at 22°C, R2 = 0.67. (TIF) [file pone.0177913.s004.tif]

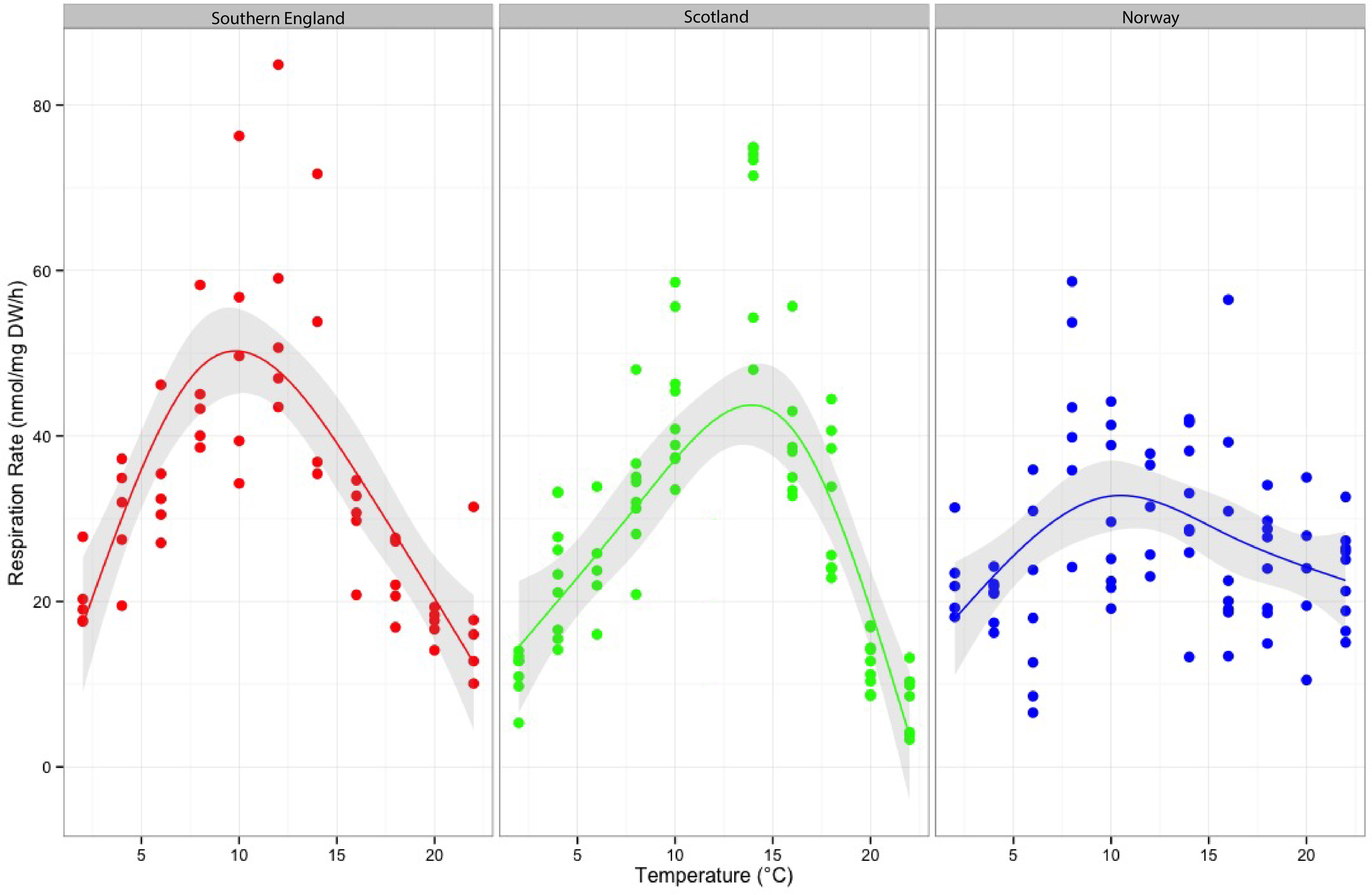

Supplement: S3 Fig — Three populations are compared: southern England (n = 54), Scotland (n = 79) and Norway (n = 81). Scatter plot plus smoothing curve (shaded area = residuals)–a line that represents the data but does not go through each data point—is displayed. Data were analysed in R. Data are available in electronic supplementary material (S1 Dataset). (TIFF) [file pone.0177913.s005.tiff]
